# The emerging double-edged sword role of exosomes in Alzheimer’s disease

**DOI:** 10.3389/fnagi.2023.1209115

**Published:** 2023-06-16

**Authors:** Tao Liang, Zubo Wu, Junjun Li, Suyuan Wu, Wuhe Shi, Lin Wang

**Affiliations:** ^1^Department of Clinical Laboratory, Union Hospital, Tongji Medical College, Huazhong University of Science and Technology, Wuhan, China; ^2^Department of Pediatrics, Union Hospital, Tongji Medical College, Huazhong University of Science and Technology, Wuhan, China; ^3^Department of Neurosurgery, Union Hospital, Tongji Medical College, Huazhong University of Science and Technology, Wuhan, China; ^4^Research Center for Tissue Engineering and Regenerative Medicine, Union Hospital, Tongji Medical College, Huazhong University of Science and Technology, Wuhan, China

**Keywords:** Alzheimer’s disease, exosomes, pathogenesis, amyloid β, tau

## Abstract

Alzheimer’s disease (AD) is the most common neurodegenerative disease characterized by progressive loss of memory and cognitive dysfunction. The primary pathological hallmarks of AD are senile plaques formed by deposition of amyloid β (Aβ) protein, intracellular neurofibrillary tangles resulting from hyperphosphorylation of microtubule-associated protein tau, and loss of neurons. At present, although the exact pathogenesis of AD is still unclear and there is a lack of effective treatment for AD in clinical practice, researchers have never stopped exploring the pathogenic mechanism of AD. In recent years, with the rise of the research of extracellular vesicles (EVs), people gradually realize that EVs also play important roles in neurodegenerative diseases. Exosomes, as a member of the small EVs, are regarded as carriers for information exchange and material transport between cells. Many cells of the central nervous system can release exosomes in both physiological and pathological conditions. Exosomes derived from damaged nerve cells can not only participate in Aβ production and oligomerization, but also disseminate the toxic proteins of Aβ and tau to neighboring neurons, thereby acting as “seeds” to amplify the toxic effects of misfolded proteins. Furthermore, exosomes may also be involved in the degradation and clearance process of Aβ. There is increasing evidence to suggest that exosomes play multiple roles in AD. Just like a double-edged sword, exosomes can participate in AD pathology in a direct or indirect way, causing neuronal loss, and can also participate in alleviating the pathological progression of AD. In this review, we summarize and discuss the current reported research findings on this double-edged role of exosomes in AD.

## 1. Introduction

Alzheimer’s disease (AD), also known as senile dementia, is one of the most prevalent central neurodegenerative diseases. AD mainly shows the gradual loss of self-care ability, progressive memory decline and behavioral cognitive impairment, accompanied by neuropsychiatric abnormalities, which seriously affect the quality of life of AD patients (Si et al., 2023). With the aging of the global population becoming increasingly prominent, the number of cases of AD is increasing year by year, which has become a major public health problem in front of us, and brought a heavy burden to individuals, society and families. Although the researchers have invested a great deal of financial resources and manpower to explore the pathogenesis of AD, it has not been fully elucidated, and to date, there is still no effective treatment available to stop the development of AD. Currently, with the rising and deepening of the research of extracellular vesicles (EVs), people have gradually realized that a kind of small EVs called exosomes to play a crucial role in AD pathogenic process (Gomes et al., 2022).

Exosomes are EVs with a phospholipid bilayer membrane structure. Exosomes can be released from most cell types and are widely present in biological fluids. Although exosomes containing specific cargoes are closely related to their parent cells of origin, they mainly include proteins, lipids, and nucleic acid molecules. Exosomes transport these cargoes from the donor cells to neighboring or more distant recipient cells, which can internalize exosomes from the extracellular space via several mechanisms including phagocytosis, pinocytosis, endocytosis or plasma membrane fusion (Zhang et al., 2021). In addition, exosomes are considered as a carrier tool that can move dynamically between cells and play an important role in material transfer and information exchange. Studies have shown that many cells in the central nervous system, including neurons, astrocytes, oligodendrocytes, microglia, and endothelial cells, can release exosomes and play both beneficial and pathogenic roles in physiological and pathological conditions (Kanninen et al., 2016). It has been recognized that the two typical pathological features of AD are senile plaques formed by the deposition of extracellular amyloid β (Aβ) protein and intracellular neurofibrillary tangles formed by the hyperphosphorylation of microtubule-related protein tau (d’Errico and Meyer-Luehmann, 2020). There is increasing experimental evidence showing that exosomes play indispensable roles in the formation and dissemination of these two major pathological hallmarks (Soliman et al., 2021). More importantly, studies have found that exosomes can play a double-edged role in AD. On the one hand, exosomes derived from damaged nerve cells can transfer amyloid precursor protein (APP), γ/β-secretase, Aβ peptide, C-terminal APP and tau protein to adjacent healthy neurons to accelerate the death of peripheral neurons, thus leading to the spread of pathological features of AD (Perez-Gonzalez et al., 2012; Sardar Sinha et al., 2018). On the other hand, exosomes also have a positive role in promoting Aβ clearance. For example, neuron-derived exosomes (NDEs) can transport Aβ to the lysosomes of microglia and degrade in the lysosomes (Yuyama et al., 2012). In short, the roles of exosomes have been attracted much attention in AD. Many previous reviews have mainly illustrated the clinical value of exosomes in serum, plasma, urine or cerebrospinal fluid as a diagnostic tool in the early diagnosis of AD. However, the main focus of our study is to summarize the multiple roles of exosomes in the production, transport, dissemination and clearance of AD toxic proteins in order to provide new ideas for the pathogenesis, targeted therapy and early diagnosis of AD.

## 2. Biological characteristics of exosomes

**FIGURE 1:**
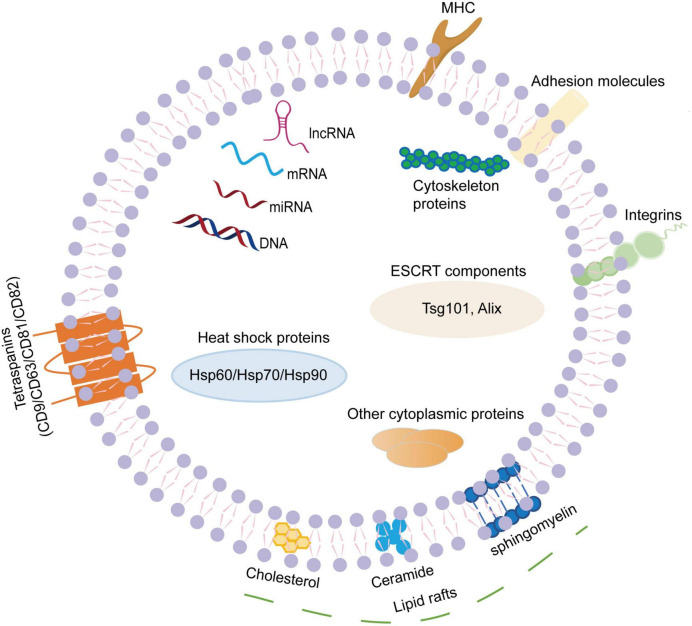
The composition of exosomes. Exosomes are small extracellular vesicles with a diameter of 30–150 nm. The exosome membranes are mainly decorated with various lipids and proteins. The lipids mainly present in the lipid raft regions, including ceramide, cholesterol, sphingomyelin, phospholipids, glycerol phospholipids, etc. The surface of exosome contains specific transmembrane molecules (CD9, CD63, CD81, and CD82), adhesion proteins, integrins, MHC, etc. In addition, exosomes can pack a wide variety of cargoes, including nucleic acids (miRNAs, mRNA, DNA, lnc RNA, etc.), proteins (Alix, TSG101, heat shock proteins, cytoskeletal proteins, etc.). MHC, major histocompatibility complex; mRNA, messenger RNA; miRNA, microRNA; lnc RNA, long non-coding RNA; TSG101, tumor susceptibility gene 101.

Exosomes are small EVs with a diameter of 30-150nm. They can be widely isolated from a variety of body fluids, such as blood, cerebrospinal fluid, saliva, thoracic and abdominal fluids, urine, semen, breast milk, etc (DeLeo and Ikezu, 2018; Pascual et al., 2020). Exosome membranes are mainly composed of phospholipids and proteins. The surface of exosomes contains specific marker molecules (CD9, CD63, CD81, CD82, adhesion proteins, integrins, glycoproteins, etc.), and exosomes can pack a wide variety of cargoes, including but not limited to nucleic acids (microRNAs, mRNA, DNA, ribosomal RNA, long non-coding RNAs, etc.), proteins (Alix, tumor susceptibility gene 101 protein, heat shock proteins, lipid-associated proteins, cytoskeletal proteins, etc.) and lipids (lipid raft-associated lipids, ceramides, sphingolipids, phospholipids, glycerol phospholipids, etc.) (Song et al., 2020; Figure 1). Although the cargoes in exosomes are similar to some extent, they also have some cell specificity, which is related to the specific substances contained in the parent cells of their origins and the physiological or pathological conditions of exosome production. Additionally, exosome cargoes can change under different stimulus factors, and can be detected by proteomics technology, lipidomics technology, nucleic acid sequencing and digital PCR (Kanninen et al., 2016).

In view of the biological characteristics of exosomes, the value of exosomes as biomarkers to distinguish healthy and disease states has attracted more and more attention. As a tool for disease diagnosis, exosomes have many unique advantages. Firstly, exosomes can be extracted from majority of body fluids in a convenient and non-invasive way, thus avoiding the damage to the organism caused by sampling. Secondly, exosomes are rich in cargoes, and their membrane structure can protect the cargoes from degradation by external factors, thereby maintaining their integrity and biological activity. Moreover, the exosome cargoes vary with the disease state and are closely related to the process of disease. Remarkably, exosomes can appear in peripheral blood by crossing the blood-brain barrier via bilayer lipid structure. Considering the prevalence of blood samples, researchers pay more attention to search for disease-specific biomarkers from blood exosomes. However, due to the widespread and complex sources of blood exosomes, the extracted total exosomes can not accurately reflect the disease status of the nervous system, and brain-derived exosomes (BDEs) need to be further isolated from blood exosomes.

## 3. Brain-derived exosomes

BDEs are a group of exosomes secreted by central nervous system cells, including neuron-derived exosomes (NDEs) and astrocyte-derived exosomes (ADEs). By detecting the changes of BDEs contents, the intrinsic pathological changes of brain can be indirectly reflected. This is not only to better explore the pathogenesis of exosomes in AD, but also to provide a minimally invasive method for the early diagnosis of AD, which is considered as a form of “liquid biopsy” (Li et al., 2019). A precipitate/immunoaffinity system has been developed at an earlier stage for the isolation of NDEs from peripheral blood. NDEs were first described in 2015. After extracting total plasma exosomes using ExoQuick exosome precipitation solution, NDEs are further isolated using antibodies against neuronal cell adhesion molecules (Fiandaca et al., 2015). Neuronal L1 cell adhesion molecule (L1CAM) or neural cell adhesion molecule (NCAM) can be used as a biomarker for isolating BDEs. This BDEs isolation technique has also been verified and used in other study (Goetzl et al., 2016a). In the same year, their experimental team took a similar approach with antibodies against glutamine aspartic acid transporters to enrich ADEs in peripheral blood (Goetzl et al., 2016b). The above innovative isolation methods of BDEs subsets have greatly triggered a research upsurge in the pathogenesis and biomarkers of central nervous system diseases. Subsequently, due to the development of experimental techniques, some other BDEs capture techniques have been gradually developed. For instance, a two-step immunocapture technique using immunomagnetic beads is established to isolate specific NCAM/amphiphysin 1 or NCAM/ATP-binding cassette transporter A1 (ABCA1) NDEs from total plasma exosomes (Li et al., 2022a,b). At present, most research strategies are to use L1CAM or NCAM antibody combined with magnetic beads to capture NDEs. Although earlier literatures have shown that L1CAM is an exosome surface marker molecule specifically derived from neurons (Fauré et al., 2006), there are some other studies have suggested that L1CAM is not only expressed in neurons, but also in other peripheral tissue cells (Fowler, 2019). Surprisingly, L1CAM has a soluble variant, which can exist in a free form in body fluids (Angiolini et al., 2019). A more recently reported study indicates that L1CAM mainly exists in the form of free protein in cerebrospinal fluid and plasma, and it may not be an ideal biomarker of NDEs (Norman et al., 2021). Thus, the extraction purity of NDEs or ADEs is crucial for the subsequent experimental results. This requires better isolation and purification techniques and further needs to discover higher specificity of BDEs marker molecules.

## 4. Exosomes: a double-edged sword in Alzheimer’s disease

**FIGURE 2:**
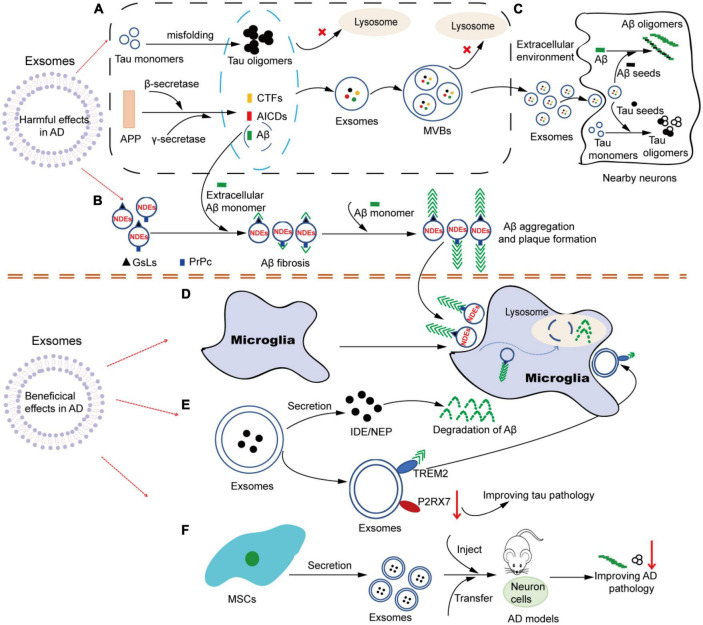
The multiple roles of exosomes in AD. **(A)** Tau and APP-related metabolites accumulate intracellular and are encapsulated into exosomes. Under pathological conditions, soluble Tau monomers can be misfolded into Tau oligomers after hyperphosphorylation in neurons. In addition, APP can be continuously cleaved by β-secretase and γ-secretase to produce APP-related products such as CTFs, Aβ and AICDs. When lysosome function is impaired, these Tau and APP-related metabolites accumulate in the cytoplasm and are easily encapsulated in exosomes. Similarly, due to dysdegradation of Tau- and Aβ-containing exosomes in lysosomes, exosomes in MVBs are filled with Tau- and APP-related products, which are released into the extracellular environment as exosomes are transported. **(B)** NDEs are involved in the oligomerization of Aβ. The surface of NDEs is enriched with GSLs and PrPC, and both GSLs and PrPC can bind to Aβ, thereby mediating the involvement of exosomes in the fibrosis and oligomerization of Aβ. Therefore, NDEs can capture Aβ via GSLs and PrPC in a single or synergistic manner, thus accelerating Aβ aggregation and amyloid plaque formation. **(C)** Exosomes in Tau and Aβ spread. Exosomes containing Tau-and APP-related metabolites released into the extracellular environment can be used as pathologic seeds to be captured by adjacent neuronal receptor cells and participate in Tau and Aβ oligomerization in the recipient cells. Similarly, infected recipient cells can further spread the pathology to other previously unaffected cells, causing the proliferation of pathological proteins from cell to cell. **(D)** Microglia are involved in NDEs-dependent Aβ clearance. NDEs-dependent Aβ oligomers can be captured by microglia and degraded in lysosomes. **(E)** Exosomes may be involved in the improvement of Aβ and Tau pathological features through the release of contents (IDE, NEP, etc.) or specific molecules on the surface (TREM2, P2RX7, etc.). **(F)** Exosomes from different sources of MSCs can be used in AD models, showing therapeutic value and alleviating AD pathology. AD, Alzheimer’s disease; APP, amyloid precursor protein; CTFs, carboxyl-terminal fragments; AICDs, amyloid intracellular domains; Aβ, amyloid β; MVBs, multivesicular bodies; NDEs, neuron-derived exosomes; GSLs, glycosphingolipids; PrPC, cellular prion protein; IDE, insulin degrading enzyme; NEP, neprilysin; TREM2, triggering receptor expressed on myeloid cells-2; P2RX7, P2X purinoceptor 7; MSCs, mesenchymal stem cells.

Although the understanding of exosome function in AD has not been fully elucidated, with the deepening and extensive research, increasing evidence supports that exosomes play multiple roles in AD. Just like a double-edged sword, exosomes can not only participate in the pathological process of AD in a direct or indirect way, causing neuronal loss, but also help in alleviating the progression of AD pathology. Based on the current research literature, we speculate that exosomes may be involved in the pathogenesis of AD in several ways. One way is that exosomes involve in the formation, oligomerization, plaque formation and clearance of Aβ. Another way is that exosomes can act as constantly moving carriers to transport toxic cargoes (such as Aβ, tau, inflammatory molecules, etc.) or beneficial cargoes (such as enkephalins, insulin-degrading enzymes, etc.) in the brain at short or long distances. Its dissemination mechanism is similar to that of prions. Additionally, both neurons and glial cells can release and take up exosomes. As a carrier of cell-to-cell communication, exosomes can mediate the interregulation between neurons and glial cells, thus participating in the development process of AD (Figure 2).

### 4.1. The pathogenic role of exosomes in AD

#### 4.1.1. Exosomes and Aβ

##### 4.1.1.1. Exosomes participate in the APP metabolic process

Aβ is a product of the sequential cleavage of the transmembrane APP by β- and γ-secretase. Normally, intracellular Aβ can be degraded through the endosomal-lysosomal pathway. However, once the impairment of this pathway (especially the dysfunction of key molecules involved in the Aβ degradation process, such as metalloproteinase endothelin-converting enzyme), it can lead to the intracellular accumulation of Aβ, thereby making it easier for Aβ and APP metabolites to be encapsulated in exosomes and released into the extracellular space via exosomes (Pacheco-Quinto et al., 2019). Many earlier reports have described the presence of APP-associated proteins and their metabolites during Aβ production in exosomes of AD cell models and AD patients. Rajendran et al. first reported the existence of APP in exosomes, and the cleavage of β-secretase occurs in the early endosome. A minute fraction of Aβ can be secreted from the cells in association with exosomes and released from APP-transfected cells into the culture medium. Furthermore, they found that the brain slices of all AD patients show the enrichment of exosome marker Alix around small neuritic plaques, thus confirming the accumulation of exosomes around the amyloid plaques (Rajendran et al., 2006). Subsequently, Vingtdeux et al. also observed the presence of APP-related metabolites APP-carboxyl-terminal fragments (CTFs) and amyloid intracellular domains (AICDs) in multivesicular bodies (MVBs) of differentiated neuroblastoma cells transfused with human APP. Their data showed that MVBs are essential organelles for APP metabolism, and all APP metabolites can be secreted into the extracellular space (Vingtdeux et al., 2007). More importantly, several key members of the secretase family of proteases (β-site APP cleaving enzyme 1, presenilin 1, presenilin 2, and disintegrin and metalloprotease 10) can also be localized in exosomes (Sharples et al., 2008). APP-related metabolites in NDEs are also toxic and exhibit similar pathological features of AD in healthy neurons. Zheng et al. injected exosomes harvested from the conditioned medium of HEK293-APP Swe/Ind cells into the hippocampal dentate gyrus via a stereotactic approach. They found that exosomes containing pathogenic proteins (full-length APP and APP metabolites) showed higher neurotoxicity and could impair neurogenesis in the hippocampus (Zheng et al., 2017a). In conclusion, considering the process of exosome formation, combined with the metabolic site and process of APP, it is not surprising that these endosome-associated protein products can be detected in neuronal exosomes under the pathological conditions of AD (Figure 2A).

In addition to the direct involvement of exosomes in APP metabolism through the above-mentioned ways, exosomes can also participate in indirect ways. A recent study reported a novel mechanism to regulate APP expression in different cells via the exosome-mediated miR-185-5p delivery. Exosomes isolated from AD mouse brains and APP-overexpressed N2a cell cultures significantly increased APP expression levels in recipient cells. Surprisingly, the effects of exosomes on APP expression in recipient cells are not mediated by the direct transferring of APP gene products. Instead, it is mediated by the reduction of the expression levels of miR-185-5p in exosomes (Ding et al., 2022). This indirect regulation has increased the complexity of exosomes involved in APP metabolism, which still needs more evidence to confirm.

##### 4.1.1.2. Exosomes promote Aβ aggregation and plaque formation

Emerging evidence suggests that exosomes can promote Aβ aggregation and then accelerate the formation of amyloid plaques. The correlation between exosomes and Aβ fibrosis is first reported in the study of Yuyama et al. In their study, they found that the assembly of Aβ is markedly accelerated when incubates with exosome portions prepared from the culture medium of PC12 cells treated with chloroquine and KCl. The formation of extracellular amyloid fibers is associated with GM1 gangliosides on exosomes, which is blocked by antibodies that recognize the GM1-Aβ complex (Yuyama et al., 2008). Moreover, it was Yuyama and his colleagues who revealed that exosomes isolated from both N2a and primary neurons promote fibrillization of Aβ40 and Aβ42 with Aβ42 aggregation occurring more rapidly. Using cholera toxin B subunit blocking of ganglioside GM1 and endoglycoceramidase-treated glycosylysis *in vitro* cell experiments, they demonstrated that glycosphingolipids (GSLs) glycochains on the surface of exosomes play an important role in inducing the Aβ fibril formation (Yuyama et al., 2012). GSLs are a group of glycochain membrane lipids that are localized on the outer layer of cells and exosomal membranes, and their glycans are exposed to the external environment. GSLs laterally traverse the cell membrane and form clusters at high densities, which monomeric Aβ is able to recognize and bind (Yuyama and Igarashi, 2022). Many studies have documented that the rich lipid rafts in GSLs (including GM1 ganglioside) can bind to Aβ and promote its aggregation (Ariga et al., 2001; Hayashi et al., 2004). Exosomes, especially NDEs, enrich GSLs. Therefore, it is speculated that exosomes may indeed act as seeds for Aβ aggregation and plaque formation.

In addition to GSLs, cellular prion protein (PrP(C)) is also highly enriched in exosomes. PrP(C) is a glycosylphosphatidylinositol-anchored surface glycoprotein that is localized on the outer leaflet of the membranes of neurons and exosomes. Studies have proved that exosomes can also bind to Aβ via PrP(C), which has the highest binding affinity for dimeric, pentameric and oligomeric Aβ species, and accelerates the fibrosis of Aβ, thereby reducing the neurotoxic effects of oligomeric Aβ (Falker et al., 2016). Taken together, these findings suggest that NDEs can capture Aβ via GSLs and PrP(C) in a single or synergistic manner, thus accelerating Aβ aggregation and amyloid plaque formation (Figure 2B).

##### 4.1.1.3. Exosomes contribute to the spread of Aβ

On the one hand, NDEs, as described above, carry proteins associated with the Aβ generation pathway, such as APP, β-site APP cleaving enzyme 1, γ-secretase and their pyrolysis products β soluble APP, α soluble APP, CTFs and Aβ (Goetzl et al., 2016b). On the other hand, exosomes isolated from the brains of APP transgenic mice show higher levels of flAPP and APP CTFs than those of wild-type mice, suggesting that APP-related metabolites can accumulate in exosomes under the pathogenic conditions of AD, thus contributing to the spread of amyloid protein between cells (Perez-Gonzalez et al., 2012). The above two factors provide some theoretical basis for the pathological propagation of exosomes involved in AD. Accumulating evidence that Aβ can be transmitted in the brain from the source cells to the recipient cells by means of exosomes as vectors, in a way similar to prion diseases (Figure 2C). Research by Sinha et al. showed that exosomes can mediate the pathologic proliferation of Aβ between cells for the first time. NDEs have been demonstrated to mediate Aβ oligomers (oAβ) diffusion between neurons by co-localization of oAβ with exosomes in cells. Most importantly, exosomes carrying oAβ can be internalized in cultured neurons and spread their toxic contents to nearby cells. Blocking exosome formation, secretion, or uptake has been found to reduce oAβ diffusion and associated toxicity (Sardar Sinha et al., 2018). Besides, exosomes isolated from the cerebrospinal fluid and plasma of AD patients, or from the medium of neural cells expressing AD presenilin 1 mutations may mediate transcellular spread of pathogenic Aβ species, disrupt neuronal Ca^2+^ homeostasis, impair mitochondrial function, and induce neuronal apoptosis (Eitan et al., 2016). Another more convincing study is that Zheng et al. injected exosomes isolated from peripheral plasma into the hippocampus of AD mouse models in order to investigate the diffusion process of exosomes, and observed that exosomes can spread from the dentate gyrus to other regions of the hippocampus and cortex, and to the entire brain (Zheng et al., 2017b). Another study showed that exosomes harvested from the conditioned medium of HEK293-APP Swe/Ind cells contain APP and are able to efficiently transfer APP to normal nerve cells, which also provides evidence for direct intercellular diffusion of amyloid proteins via exosomes (Zheng et al., 2018).

#### 4.1.2. Exosomes and tau pathology

It is known that intracellular neurofibrillary tangles formed by hyperphosphorylated tau protein are one of the typical pathological features of AD, and the pathological proliferation of tau is also a hallmark of AD. Although the toxic effects of tau occur primarily inside the cell, tau can be released into the extracellular space as a “seed”. The tau “seeds” can be taken up by other healthy recipient cells and transferred from one cell to another (Figure 2C). Hence, tau pathology is spread through different regions of the brain in a manner similar to prions. This proliferative effect significantly promotes the pathological process of tau. However, in earlier times, investigators believe that tau is passively released into the extracellular space only through cell death, and then these membrane-free tau “seeds” spread to neighboring nerve cells (Gómez-Ramos et al., 2006). On the contrary, in recent years, with the discovery of EVs, people gradually realize that exosomes can also serve as an important vehicle to actively secrete tau “seeds” into the extracellular environment. Recently, the role of exosomes in the spread of tau pathology has gradually become one of the hot issues concerned by many researchers.

Tau oligomers first pass through the endoplasmic reticulum and Golgi apparatus, then become part of vesicles and subsequently enter exosomes. Under physiological conditions, exosomes containing tau are degraded by lysosomes. However, once the lysosome is damaged, it contributes to tau accumulation in exosomes. Earlier studies have reported that some forms of tau exist in the EVs (Dujardin et al., 2014), and these EVs isolated from neuronal culture can induce abnormal tau phosphorylation in the brain of wild-type mice, showing significant neurotoxic effects (Aulston et al., 2019). Moreover, another study revealed that most of the intracellular tau is full-length, while the bulk of the extracellular tau is free-floating, unaggregated and C-terminal truncated, lacking the microtubule binding regions necessary for aggregation. Only a small number of tau is encapsulated in exosomes (Kanmert et al., 2015). These tau proteins packed in exosomes contain the microtubule binding regions and have the ability to aggregate. Furthermore, it has been confirmed that there are small amounts of full-length tau in neuronal exosomes (Guix et al., 2018). In summary, the detailed mechanisms of tau encapsulated in exosomes still need further study.

Evidence from some studies illustrates that tau aggregates can spread and replicate in the brain in a prion-like manner via exosomes. Tau oligomers delivered by exosomes may act as “seeds”, and the uptake of pathological tau “seeds” leads to tau misfolding into a toxic conformation in recipient cells (Jackson et al., 2022). Exosomes isolated from the brains of tau transgenic rTg4510 mice carry a large number of tau “seeds”, thereby inducing endogenous tau aggregation in the recipient cells and increasing tau phosphorylation and oligomer formation. This evidence confirms that EVs are the carriers of tau pathology (Baker et al., 2016; Polanco et al., 2016). Many *in vivo* experiments have demonstrated that tau-containing neuronal exosomes from AD patients can induce the formation of neurofibrillary tangles (NFT) in rodents, exhibit tau neurotoxic effects, and thus reproduce the disease characteristics triggered by the original exosome contents. Winston et al. found that NDEs extracted from the plasma of patients with mild cognitive impairment and AD are able to seed p-tau pathology and induce AD-like neuropathology in the brains of normal mice (Winston et al., 2016). In addition, another study has shown that neuronal exosomes derived from human tau have toxic effects on recipient mouse neurons *in vivo* and cause long-distance propagation of tau pathology and neurodegeneration (Winston et al., 2019). A similar phenomenon has also been observed in the study of Reilly et al. (2017). Surprisingly, further research by Polanco et al. showed that a simple neuronal circuit model was built to observe the diffusion of exosomes in the interconnected neurons using a microfluidic culture system. The findings suggest that exosomes can spread the tau protein over long distances through a novel hijacking mechanism of endosomes, thereby increasing the pathogenic potential and radius of action of the exosomes (Polanco et al., 2018). In a recent study, to reveal how tau seeds contained within internalized exosomes exploit mechanisms of lysosomal degradation to escape the endosome and induce tau aggregation in the cytoplasm, their research team also found that the enzymatic activities of lysosomes can penetrate the exosomal and endosomal membranes, thereby promoting the entry of exosomal tau seeds into the cytoplasm and inducing its aggregation (Polanco et al., 2021). In earlier studies on the relationship between exosomes and tau, several studies described that the spread of tau pathology is achieved by the direct transmission of exosomes across synapses between interconnected neurons, and the depolarization of neurons faciliates the release of tau-containing exosomes (Wang et al., 2017). Recent literatures have also reported that only exosomes and vesicle-free tau seeds are shown to propagate across synapses along neural networks (Polanco and Götz, 2022). Most of the exosomal tau released from AD synapses is C-terminal-truncated and oligomeric, and with seeding activity that is enhanced by Aβ (Miyoshi et al., 2021). These results also support the ideas that Aβ-induced pathology may directly or indirectly drive tau-mediated neurotoxicity and NFT formation (Blurton-Jones and Laferla, 2006).

It is becoming increasingly clear that microglia may play a critical role in the interneuronal transmission of tau pathology promoted by exosomes. It has been shown that microglia can spread tauopathy by internalization and secretion of exosomes. Microglia propagates tau pathology via secretion of tau-containing exosomes. The depletion of microglia or the inhibition of exosome synthesis significantly reduces tau propagation *in vitro* and *in vivo*. Surprisingly, tau propagation mediated by microglia can occur not only through trans-synaptic transmission, but also in non-trans-synaptic pathways (Asai et al., 2015). Most importantly, several reports have illustrated that some genes closely related to the risk of AD morbidity can interfere with the spread of tau pathology by influencing the production of exosomes in microglia. Bridging integrator 1 is a gene associated with late-onset AD. Overexpression of bridging integrator 1 promotes the release of tau-enriched EVs from microglia, and contributes to exacerbate tau pathology in P301S tau transgenic PS19 mice, thus promoting the spread of AD-related tau pathology (Crotti et al., 2019). Triggering receptor expressed on myeloid cells 2 (TREM2) is a transmembrane protein produced by microglia in the brain. Variations in TREM2 have been shown to increase the risk of late-onset AD (Ulland and Colonna, 2018). It has been demonstrated that Trem2 deletion can enhance the transport, distribution and seeding of tau by microglial exosomes (Zhu et al., 2022).

#### 4.1.3. Exosomes and inflammation

Increased research has focused on exosomes as important communication mediators between neurons and glial cells. Exosomes induce neuroinflammation by acting as inflammatory mediators via carrying inflammation-causing cargoes (Weng et al., 2022). The accumulation of Aβ and hyperphosphorylation of tau have been shown to continuously activate microglia and astrocytes, and promote inflammatory responses (Novoa et al., 2022). Aβ or tau can be effectively encapsulated in exosomes, and activated glial cells or neuronal cells can release exosomes into the extracellular space, thus amplifying the neuroinflammatory effects caused by toxic proteins (Gupta and Pulliam, 2014). Additionally, microRNAs, as an important nucleic acid cargo in exosomes, have been found to be involved in the induction of neuroinflammation. It has been reported that NDEs containing miR-21-5p can be phagocytosed by microglia and induce microglia M1 polarization, which leads to increased release of neuroinflammatory factors, inhibition of neurite outgrowth, increased accumulation of P-tau, and increased the apoptosis of PC12 cells (Yin et al., 2020). Another study from Wei et al. has shown that miR-182-5p delivered by plasma exosomes can target brain-derived neurotrophic factor (BDNF) and activate the nuclear transcription factor-κB pathway, thereby promoting sevoflurane-induced neuroinflammation and cognitive dysfunction in aged postoperative cognitive dysfunction rats (Wei et al., 2022). In addition to enhancing and spreading inflammation in the extracellular microenvironment, exosomes can also play an anti-inflammatory role. Recent studies have reported the anti-inflammatory therapeutic value of exosomes due to their excellent properties, such as carrier transport, ease of modification and encapsulation of therapeutic agents. Related studies have also demonstrated that the activation of nucleotide-binding oligomerization domain-like receptor pyrin domain-containing 3 (NLRP3) inflammasome is closely associated with the occurrence of AD (Liang et al., 2022). By blocking the assembly of NLRP3 inflammasome, the exosome-like nanoparticles from ginger rhizomes strongly inhibit the downstream pathways of NLRP3 inflammasome activation, including caspase1 autocleavage, interleukin 1β and interleukin 18 secretion, and pyroptotic cell death (Chen et al., 2019). Under some specific conditions, exosomes released from microglia can also exert an anti-inflammatory role. For instance, the increased miR-124-3p in microglial exosomes promotes the anti-inflammatory M2 polarization of microglia, inhibits neuronal inflammation, and transfers into neurons to promote neurite outgrowth during traumatic brain injury (Huang et al., 2018). Taken together, whether exosomes promote or inhibit the inflammatory response may be related to the different stimulants and the cell types that produce exosomes, and further research is needed to explore the potential mechanism.

### 4.2. The protective role of exosomes in AD

#### 4.2.1. NDEs contribute to the Aβ clearance process mediated by glial cells

In contrast, while accumulated evidence has demonstrated that NDEs can be involved in the formation and spreading of the pathological features of AD, some studies have confirmed that NDEs also exert a protective role in the progression of AD. Therefore, NDEs play a dual role. Growing evidence indicates that NDEs and glial cell-derived exosomes may participate in Aβ clearance through independent or synergistic action and transfer neuroprotective substances between cells to alleviate the nervous system injury (Figure 2D). Earlier studies have shown that exosomes released from neuroblastoma N2a can capture and bind to Aβ via GSLs on their surface and be transported into microglia for degradation. Moreover, *in vivo* studies further provide evidence that the infusion of NDEs into the brains of APP mice can reduce Aβ levels and ameliorate Aβ-related pathologies (Yuyama et al., 2014). The next year, further research demonstrated that neuronal exosomes, but not glial exosomes, have abundant GSLs and can capture Aβ. Infusion of neuronal exosomes into the brains of APP transgenic mice can reduce Aβ and amyloid deposition, and these findings highlight the role of neuronal exosomes capable of capturing Aβ as messengers for Aβ clearance (Yuyama et al., 2015). All the above evidence have clearly demonstrated that intracerebral NDEs administration can improve the Aβ-related pathogenesis, which may provide a new direction for the treatment of AD. Furthermore, Yuyama et al. investigated that NDEs can drive the conformational changes of Aβ through GSLs on their surface. The changes in Aβ conformation can serve as seed on which other Aβ combines to form Aβ oligomers, and then form amyloid fibrinates. The Aβ-bound exosomes are endocytosed into endosomal-lysosomal system by microglia in a phosphatidylserine-dependent manner and promote the degradation of Aβ. Moreover, Yuyama et al. further demonstrated that the secretion of NDEs is modulated by the activity of sphingomyelin metabolic enzymes, including nSMase2 and sphingomyelin synthase 2. Inhibition of nSMase2 prevents exosome production, while inhibition of sphingomyelin synthase 2 promotes exosome release (Yuyama and Igarashi, 2022). In the co-culture experiment of N2a cells transfected with human APP and microglia cells, inhibition of sphingomyelin synthase 2 activity to improve the secretion of neurons’ exosomes can enhance the uptake of Aβ by microglia cells, and significantly decrease the level of extracellular Aβ (Yuyama et al., 2012). However, contrary to the results of this study, Dinkins et al. revealed that ADEs can also promote Aβ accumulation, but interfere with the uptake of Aβ by glial cells, thus promoting the formation of plaques *in vivo*. Inhibition of SMase2 activity by GW4869 or 5 × FAD mouse model targeted at nSMase2 gene deletion blocks exosome synthesis *in vivo* and *in vitro*, decreases the levels of Aβ1-42, alleviates amyloid plaque load in the brain of mice, and improves the cognitive deficits (Dinkins et al., 2014, 2016). These results suggest that neurons and astrocyte-derived exosomes may differ in glial Aβ clearance, but the underlying mechanisms remain unclear.

#### 4.2.2. Microglia-derived exosomes are involved in the remission of AD pathology

The evidence suggests that microglia and microglia-derived exosomes also play active roles in the mechanisms of Aβ clearance or tau pathologic protection. Earlier researches have shown that microglia exosomes contribute to the secretion of insulin degrading enzyme, which is an important enzyme for the extracellular degradation of Aβ (Tamboli et al., 2010). According to a recent study, triggering receptor expressed on myeloid cells-2 (TREM2), expressed on the membrane of microglia exosomes, can mediate the secretion of exosomes. Free microglia exosomes can bind to Aβ through TREM2, releasing chemokines to alter the inflammatory levels surrounding Aβ and promote the recognition and phagocytosis of Aβ by microglia cells (Huang et al., 2022). In addition to its roles in Aβ clearance, TREM2 can also affect tau pathology. Trem2 deletion, as previously described, can enhance tau trafficking, distribution, and pathological spread through microglia exosomes (Jain and Ulrich, 2022; Zhu et al., 2022). Conversely, upregulation of TREM2 in microglia inhibits the inflammatory response and ameliorates the pathological effects of activated microglia on neuronal tau hyperphosphorylation (Jiang et al., 2018). These evidence confirm that TREM2 can be involved in protecting tau pathology via microglia exosomes. P2X purinoceptor 7 (P2RX7) is an ATP-gated cationic channel, and its enrichment in microglia promotes exosome secretion. Administration of the P2RX7-specific inhibitor GSK1482160 to P301S tau mice significantly reduces the number of exosomes and accumulation of tau in the hippocampus, thereby improving working and contextual memory of model mice (Ruan et al., 2020). Inhibition of P2RX7 to down-regulate exosome expression can also be regarded as a novel pathway to improve tau pathology (Figure 2E).

#### 4.2.3. The therapeutic role of stem cell-derived exosomes in AD

It is necessary to take into consideration that recent studies have focused on the therapeutic effects of exosomes in AD, which further demonstrates the beneficial effects of exosomes. In this review, the therapeutic values of exosomes from different sources in AD are summarized in Table 1. However, it should be noted that exosomes that play protective roles in these studies are mainly from healthy cells. At present, numerous reports have described that stem cell-derived exosomes may play a positive role in alleviating the pathological features of AD (Figure 2F). Research by Lee et al. showed that exosomes secreted by adipose-derived stem cells reduce β-amyloidosis and neuronal apoptosis in AD transgenic mouse models and enhance the axon growth in the brain of AD patients (Lee et al., 2018). Further study indicates that mesenchymal stem cell (MSC)-derived exosomes can also significantly improve the pathological and cognitive deficits of AD (Chen et al., 2021). Exosomes derived from bone-MSCs alleviate cognitive decline in AD-like mice by improving BDNF-associated neuropathology (Liu et al., 2022). In addition, double transgenic APP/PS1 mice injected with bone-MSCs can activate the sphingosine kinase-1/sphingosine-1-phosphate signaling pathway to reduce Aβ deposition and promote cognitive function recovery in AD mice (Wang and Yang, 2021). Exosomes isolated from human umbilical cord MSCs (hucMSCs) have been shown to have therapeutic effects in many inflammation-related diseases. Ding et al. observed that hucMSC-exosomes injection contributes to the removal of Aβ deposition, alleviates cognitive dysfunction in AD model mice, and regulates the activation of microglia cells, thus alleviating neuroinflammation (Ding et al., 2018). Katsuda et al. also found that human adipose tissue-derived MSCs (ADSCs) secrete exosomes carrying the enzyme activity neprilysin (NEP). NEP is an enzyme that is important for the extracellular degradation of Aβ. The ADSCs-derived exosomes are transferred into N2a cells and reduced intracellular Aβ levels in N2a cells (Katsuda et al., 2013, 2015). In addition to stem cells, some other cell-derived exosomes or bioengineered exosomes also play beneficial roles in AD. Pan et al. used human brain microvascular endothelial cells derived exosomes inheriting p-glycoprotein as an Aβ cleansing system to remove Aβ peptides from the brain by specific capture between p-glycoprotein and Aβ, which can facilitate the clearance of Aβ and effectively ameliorate the cognitive dysfunction of AD mice (Pan et al., 2020). Moreover, M2 microglia-derived exosomes may also play a protective role in the pathogenesis of AD by improving PINK1/Parkin-mediated mitophagy (Li et al., 2022a). Based on bioengineering technology, microglia-targeting exosomes and targeted drug delivery can be designed. Mannose-modified exosomes containing gemfibrozil can not only bind with Aβ, but also specifically target microglia, thereby promoting the entry of Aβ into microglia, activating lysosome activity by exosome gemfibrozil, and accelerating lysosome-mediated clearance of Aβ in microglia (Hao et al., 2022).

**TABLE 1:** Protective roles of exosomes from different sources in AD.

Source of exosomes	Investigation model	Mechanism	References
ADSC	NSCs from the brains of TG2576 AD mice	Reducing Aβ42, Aβ40 levels, the cell apoptosis of AD neuronal cells and promoting the neurite growth.	Lee et al., 2018
	N2a cell line	Carrying enzymatically active neprilysin to decrease Aβ levels.	Katsuda et al., 2013, 2015
MSC	Human neural cell culture model with FAD mutations and AD transgenic mice	Reducing Aβ expression, restoring the expression of neuronal memory/synaptic plasticity-related genes in the cell model and improving brain glucose metabolism and cognitive function in AD mice.	Chen et al., 2021
BMSCs	A sporadic AD mouse model	Improving AD-like behaviors, inhibiting the hyperactivation of microglia and astrocytes, and improving BDNF-related neuropathology.	Liu et al., 2022
	APP/PS1 mice	Improving spatial learning and memory ability of APP/PS1 mice, enhancing the expression of SphK1 and S1P, inhibiting the levels of amyloid and promoting the expression of NeuN.	Wang and Yang, 2021
HucMSCs	AβPP/PS1 transgenic mice	Repairing cognitive disfunctions, clearing Aβ deposition, and alleviating neuroinflammation.	Ding et al., 2018
HBMVECs	Aβ-induced AD mice model	Facilitating the cerebral clearance of Aβ and ameliorating cognitive dysfunction.	Pan et al., 2020
M2 microglia	Neuronal HT-22 cells and APP/PS1 mice	Ameliorating PINK1/Parkin-mediated mitophagy	Li et al., 2022a
MExo-Gem	BV2 cells and Aβ_1–42_-induced AD mice	Binding with Aβ and specifically target microglia, thus promoting lysosome-mediated clearance of Aβ and improving the learning and memory ability of AD mice.	Hao et al., 2022

AD, Alzheimer’s disease; Aβ, amyloid beta; ADSC, adipose-derived stem cells; NSCs, neuronal stem cells; MSC, mesenchymal stem cell; FAD, familial AD; BMSCs, bone-marrow mesenchymal stem cells, BDNF, brain-derived neurotrophic factor; SphK1, sphingosine kinase-1; S1P, sphingosine-1-phosphate; HucMSCs, human umbilical cord mesenchymal stem cells; HBMVECs, human brain microvascular endothelial cells, PINK1, PTEN-induced putative kinase1; MExo-Gem, mannose-modified exosomes laden with Gem.

## 5. Conclusions and future perspectives

In recent years, the research of exosomes is gradually becoming a new hotspot and receiving more and more attention. As a kind of widespread EVs, exosomes carry a variety of cargoes and are involved in the transport of substances, information transmission and cell-to-cell regulation, playing key roles in physiological and pathological conditions. Exosomes are also now known to play a critical role in the occurrence and development of AD. This review focuses on summarizing the “double-edged sword” role of exosomes in the pathogenesis of AD. In AD, exosomes can not only promote the production of pathological proteins, the formation of aggregates and the diffusion in the brain, but also enhance the inflammatory response to accelerate the pathological process. Meanwhile, exosomes can also alleviate the pathological process by promoting the clearance of Aβ or tau. In particular, given the complexity and multiplicity of exosome interactions between cells, more evidence is needed to reveal the roles of exosomes in AD in the future, especially imaging labeling techniques that provide dynamic real-time visualization of the pathogenesis of exosomes in cells or in animal models. In addition, exosomes may serve as potential biomarkers for AD due to their unique biological characteristics. Moreover, the regulation of exosome production or the modification of exosomes as biological carriers of specific molecules may become a new therapeutic measure for AD. However, the important factors limiting the basic research and clinical application of exosomes are the lack of more specific BDEs isolation and extraction technologies, the long time consumption and lack of unified and standardized extraction process, which requires extensive and in-depth researches in these aspects in the future. Last but not least, although it is a great interest to research the detrimental or beneficial roles of exosomes in the progression of AD, the current literature has generally avoided the potential mechanisms by which exosomes exert these varying functions. For example, is it specific surface proteins that prompt exosomes to spread Aβ or tau seeds between neuronal cells, rather than carrying them to microglia for degradation? In addition, the relationship between NDEs and Aβ shows multiple functions, including participating in the oligomerization of Aβ and playing a role in the clearance of Aβ via microglia cells. Whether there is a potential regulatory role in the seemingly contradictory relationship between them remains unknown. These questions may be a new direction to explore the roles of exosomes in AD in the future, which also requires further study by researchers.

## Author contributions

TL designed, collected literatures, and wrote the manuscript. JL, WS, and SW collected some literatures. ZW and LW revised the article. All authors contributed to the article and approved the submitted version.
